# The Timing of Infarction Pain in Patients with Acute Myocardial Infarction after Previous Revascularization

**DOI:** 10.1100/tsw.2008.88

**Published:** 2008-06-13

**Authors:** Predrag M. Mitrovic, Branislav Stefanovic, Zorana Vasiljevic, Mina Radovanovic, Nebojsa Radovanovic, Gordana Krljanac, Dubravka Rajic, Predrag Erceg, Vladan Vukcevic, Ivana Nedeljkovic, Miodrag Ostojic

**Affiliations:** Division of Emergency Cardiology of the University, Institute for Cardiovascular Diseases, Clinical Center of Serbia, 11000 Belgrade, 8 Koste Todorovica, Serbia

**Keywords:** circadian rhythm, acute myocardial infarction, revascularization

## Abstract

Circadian variation of onset of acute myocardial infarction (AMI) has been noted in many studies, but there are no data about subgroups of patients with previous coronary artery bypass grafting (CABG). Because of abnormalities in the circadian rhythm of autonomic tone after surgery, it was very interesting to analyze the circadian patterns in the onset of symptoms of AMI in various subgroups of 1784 patients with previous CABG. As in the other studies, a peak occurred in the morning hours with 26.3% of the patients, but there was a second nearly equal, but higher, peak (26.4%) in the evening hours. The subgroups with specific clinical characteristics exhibited different patterns that determined these peaks in all populations. In patients older than 70 years of age, in both sexes, in smokers, diabetics, in patients with hypertension, in those undergoing beta-blocker therapy, and in patients without previous angina, two nearly equal peaks were observed, with higher evening peaks, except in those patients with hypertension and without angina. Only one peak in the evening hours was observed in a subgroup of patients with previous congestive heart failure (CHF) and non-STEMI. The subgroup of patients with previous angina and previous AMI exhibited no discernible peaks. The distribution of time of onset within the four intervals was not uniform, and the difference was statistically significant only for patients undergoing beta-blocker therapy at time of onset (*p* = 0.0013), nonsmokers (*p* = 0.0283), and patients with non-STEMI (*p* = 0.0412). It is well known that patients with AMI have a dominant morning peak of circadian variation of onset. However, analyzing a different subgroup of patients with AMI after previous CABG, it was found that some subgroups had two peaks of onset, but a higher evening peak (patients older than 70 years of age, smokers, diabetics, and a group of patients who were taking beta-blocker therapy). This subgroup of patients, together with the subgroups of patients with a dominant evening peak (patients with CHF and those with non-STEMI) and with patients with no peak (patients with previous angina and previous AMI), probably appear to modify characteristic circadian variation of infarction onset, expressing a higher evening peak, respectively to the previous CABG, with adverse consequences for central nervous system functioning.

## INTRODUCTION

A circadian variation of onset of acute myocardial infarction (AMI) has been described in many studies[1,2,3,4], but there are no data about a subgroup of patients with AMI after previous coronary artery bypass grafting (CABG). Patients with previous CABG represent a substantial percentage of the total population of patients with AMI[5]. The onset of AMI varies throughout the day, with a peak in the morning hours and a trough at night[3,4,6,7,8,9,10], but some hypotheses suggest that various mixtures of triggering mechanisms may account for thrombosis and the onset of AMI may be the result of the simultaneous occurrence of several events[11]. Some studies show that cardiac surgery has been associated with adverse consequences for central nervous system functioning[12,13]. Therefore, the purpose of the present study was to analyze if there are any differences in daily-time expression of chest pain in infarcted patients with previous cardiac surgery compared with patients without previous cardiac surgery.

## MATERIALS AND METHODS

The dates and times of symptom onset were recorded for 1784 patients with previous CABG (13.8% women, mean age 64.8 ± 8.0 years) who were recently determined to have sustained a definite AMI. One criterion for inclusion in the study was admission to the coronary care unit within 24 h of symptom onset. The patients with early perioperative AMI (within 30 days after operation) were excluded from the study. These AMIs were treated as complications of the cardiac surgery. To verify eligibility, the date and time of symptom onset and coronary care unit admission were noted as precisely as possible. The diagnostic criteria and coronary care unit protocol were identical for all patients. For inclusion, patients were required to have chest pain with verification by diagnostic electrocardiographic (ECG) changes (new ST-elevation >0.2 mV in two contiguous leads and >0.1 mV in two contiguous other leads, ST in leads depression >0.1 mV in two contiguous leads in the absence of QRS confounders, new left bundle branch block, or dynamic T wave inversion in more than one lead), cardiac enzyme changes (troponin T measurement >0.01 μg/l or a creatine kinase measurement more than twice the upper range of normal for the measuring laboratory)[14], and the ability to complete a structured interview. Interviewers used a structured data abstraction and questionnaire form. Information collected from each interview and chart review included patient age, sex, medical history, and medication use. The patient-reported time that the discomfort began was used as the onset time for AMI.

Subgroups of patients were examined, based on the following variables: age, gender, history of congestive heart failure (CHF), previous angina pectoris, previous AMI, hypertension, smoking, diabetes mellitus, use of beta-blockers before onset of symptoms, and infarction type (AMI with ST segment elevation, STEMI or AMI without ST segment elevation, non-STEMI). In addition, four 6-h intervals were defined as follows: 0:01 A.M. to 6:00 A.M., 6:01 A.M. to 12:00 P.M., 0:01 P.M. to 6:00 P.M., and 6:01 P.M. to 12:00 A.M. The presence of circadian variation was tested using the chi-square one degree of freedom goodness-of-fit test for uniform distribution. Difference in the circadian variation in the onset of AMI was assessed using a Pearson chi-square test. The ratios of the highest peak to the average of the percentages for remaining periods were computed for the entire patients and subgroups.

## RESULTS

The frequency of symptom onset for all 1784 patients is shown in Fig. 1. The distribution is not uniform ($p$ = 0.0026) and exhibits two peaks: the first peak (26.3%) was registered between the hours of 6:01 A.M. to 12:00 P.M. and the second peak (26.4%) was registered between 6:01 P.M. and 12:00 A.M. The second peak was 1.1 times the average of the other three time periods.

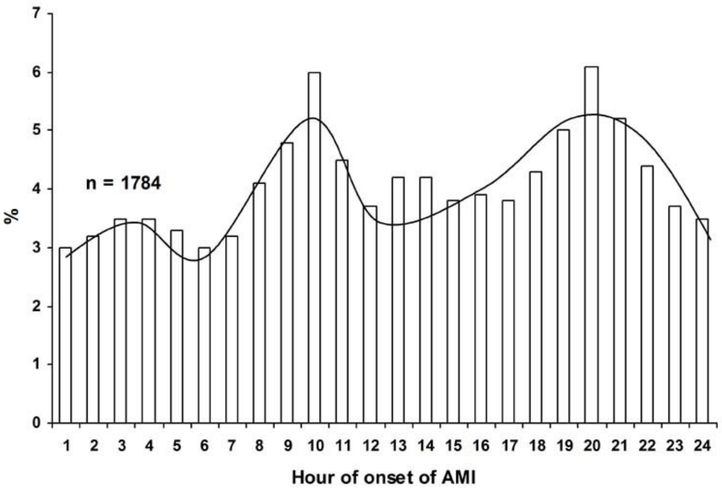

**FIGURE 1**. Distribution of hour of symptom onset of AMI after previous CABG.

We present tables that show the distribution of the time of AMI onset in 6-h intervals, according to the various characteristics of patients with previous revascularization. Two peaks (morning and evening peaks) in AMI symptom onset (Table 1) were seen among patients older than 70 years of age, in both sexes (male and female), in smokers, diabetics, in patients with hypertension, in those taking beta-blocker therapy at time of onset, and in patients without previous angina. All these groups, except those with hypertension and without previous angina, had higher evening peaks. However, only the group of patients with beta-blocker therapy at the time of onset had a statistically significant different distribution of the time of AMI onset in 6-h intervals ($p$ = 0.01).

Table 1. Circadian variation of AMI after previous CABG in patients with two peaks in the four 6-hour intervals of symptom onset

| | | Time interval | | | | |
|---|---|---|---|---|---|---|
| Two peaks | No Patients (N=1784) | 0:01 A.M. – 6:00 A.M. | 6:01 A.M.- 12:00 P.M. | 0:01 P.M.- 6:00 P.M. | 6:01 P.M.- 12:00 A.M. | p |
| Age > 70 yrs | 184 | 22.8 | 25.8* | 24 | 27.4* | 0.82 |
| Sex (M/F) | 1557/227 | 22.8/22.1 | 26.2*/27* | 24.2/23 | 26.8*/27.9* | 0.09/0.52 |
| Smoking | 266 | 22.6 | 26.2* | 24.4 | 26.8* | 0.76 |
| Diabetes mellitus | 475 | 23.8 | 27.8* | 20.4 | 28* | 0.05 |
| Hypertension | 605 | 22 | 27* | 24.6 | 26.4* | 0.29 |
| Beta-blockers therapy | 1463 | 21 | 25.8* | 25 | 28.2* | 0.01 |
| No previous AP | 1019 | 22.4 | 27.2* | 24.2 | 26.2* | 0.13 |

*) peak periods of symptom onset; AP = angina pectoris

One peak (morning peak) in AMI symptom onset (Table 2) was seen among patients 70 years of age or younger, in nonsmokers, in patients with STEMI, and in patients without diabetes mellitus, hypertension, beta-blocker therapy, CHF, or previous AMI. Subgroups with dominant evening peaks were groups of patients with CHF and patients with non-STEMI. In a subgroup of patients with previous history of CHF, 28.7% of these patients experienced the onset of AMI from 6:01 P.M. to midnight. Patients

Table 2. Circadian variation of AMI after previous CABG in patients with one peak in the four 6-hour intervals of symptom onset

| One peak | No Patients (N =1784) | Time interval 0:01 A.M. – 6:00 A.M. | 6:01 A.M.- 12:00 P.M. | 0:01 P.M.- 6:00 P.M. | 6:01 P.M.- 12:00 A.M. | p |
|---|---|---|---|---|---|---|
| Morning peak | | | | | | |
| Age ≤ 70 yrs | 1600 | 22.4 | 26.2* | 25.2 | 26.2 | 0.10 |
| No smoking | 1518 | 22.6 | 27.8* | 24 | 25.6 | 0.02 |
| No diabetes mellitus | 1309 | 22.8 | 26.2* | 25.2 | 25.8 | 0.30 |
| No hypertension | 1179 | 22.5 | 26.6* | 25.1 | 25.8 | 0.21 |
| No beta-blockers therapy | 321 | 22.6 | 27.6* | 24.8 | 25 | 0.65 |
| No CHF | 1449 | 22 | 26.4* | 25.9 | 25.7 | 0.06 |
| STEMI | 642 | 23 | 27* | 24.5 | 25.5 | 0.53 |
| No previous AMI | 965 | 22.7 | 26.4* | 25 | 25.9 | 0.37 |
| Evening peak | | | | | | |
| CHF | 335 | 22.9 | 24.6 | 23.8 | 28.7* | 0.45 |
| Non-STEMI | 1142 | 22 | 24.8 | 25.2 | 28* | 0.04 |

*) peak periods of symptom onset; CHF = congestive heart failure; STEMI = acute myocardial infarction with ST segment elevation; AMI = acute myocardial infarction; Non-STEMI = acute myocardial infarction without ST segment elevation with non-STEMI exhibited only an evening peak (28%). However, only the group of nonsmokers ($p$ = 0.02) and patients with non-STEMI ($p$ = 0.04) had a statistically significant different distribution of the time of AMI onset in 6-h intervals.

The subgroup of patients with a history of previous AMI and previous angina exhibited no discernible peak (Table 3).

The distribution of time of onset within the four intervals was not uniform and the difference was statistically significant for patients undergoing beta-blocker therapy at time of onset ($p$ = 0.0013), nonsmokers ($p$ = 0.02), and patients with non-STEMI ($p$ = 0.04) (Table 4).

## DISCUSSION

It is well known that a surge in sympathetic activity and vagal withdrawal in the morning hours alters homodynamic forces and may cause atherosclerotic plaques to rupture in the morning[15]. Also, a morning increase in platelet reactivity may make a thrombus more likely to grow and cause symptoms[16]. Many studies[2,6,7,8,9,10,17,18,19] found that the incidence of symptom onset is most frequent in the late morning, from 6:01 A.M. to noon, but in our population of patients with previous revascularization, there is also a second, higher peak in the late evening, between 6:01 P.M. and midnight. Two peaks of circadian variation of the AMI onset and resistance to thrombolysis were reported in the study of Kono et al.[4].

It has been demonstrated that patients who received beta-blocker therapy failed to exhibit the morning peak in the incidence of AMI[21,22]. Woods et al.[3] reported that in patients who had been taking a beta-blocker or calcium antagonist as a prior antianginal treatment, the 24-h cycle of onset of AMI was absent. Expression of an evening peak in patients with beta-blockers at the time of AMI is in agreement with findings in the MILIS study reported by Muller et al.[8]. Patients receiving beta-blocker therapy (n = 1463) had the highest incidence of infarction onset in the late evening (28.2%) as in the MILIS population[10].

Table 3. Circadian variation of AMI after previous CABG in patients with no peak in the four 6-hour intervals of symptom onset

| No peak | No Patients (N = 1784) | Time interval 0:01 A.M. – 6:00 A.M. | 6:01 A.M.- 12:00 P.M. | 0:01 P.M.- 6:00 P.M. | 6:01 P.M.- 12:00 A.M. | p |
|---|---|---|---|---|---|---|
| Previous AP | 765 | 24.9 | 25 | 24.9 | 25.2 | 0.99 |
| Previous AMI | 819 | 25 | 25.3 | 24.2 | 25.5 | 0.95 |

*) peak periods of symptom onset; AP = angina pectoris; AMI = acute myocardial infarction

Table 4. Circadian variation of AMI after previous CABG in subgroups of patients with significantly different 6-hour intervals of symptom onset

| | No Patients (N = 1784) | Time interval 0:01 A.M. – 6:00 A.M. | 6:01 A.M.- 12:00 P.M. | 0:01 P.M.- 6:00 P.M. | 6:01 P.M.- 12:00 A.M. | p |
|---|---|---|---|---|---|---|
| Beta-blockers therapy | 1463 | 21 | 25.8* | 25 | 28.2* | 0.01 |
| No smoking | 1518 | 22.6 | 27.8* | 24 | 25.6 | 0.02 |
| Non-STEMI | 1142 | 22 | 24.8 | 25.2 | 28* | 0.04 |

*) peak periods of symptom onset; Non-STEMI = acute myocardial infarction without ST segment elevation

The Reinfarction Study[2] showed that in patients with non-STEMI (n = 540), the incidence of infarction was higher during the last two quarters of the day (each 27%) than in the first two quarters (21 and 24%). In our population with non-STEMI (n = 1142), there was only an evening peak (28%) of onset expression, as in the study reported by Hjalmarson et al.[23]. They showed an evening peak (28.1%) in a subgroup of patients with non-STEMI. The findings of an altered circadian pattern for patients older than 70 years of age (men and women), patients with previous AMI, and smokers are at odds with the results reported in the MILIS study, as well as the findings of two peaks (morning and evening) for patients with diabetes and hypertension, and only one evening peak for patients with history of CHF, also reported in the study of Hjalmarson et al. The subgroup of revascularized patients with previous angina exhibited the typical circadian rhythm with no peak and requires confirmation in further studies.

## CONCLUSIONS

Analyzed subgroups of patients older than 70 years of age, smokers, diabetics, and the respected group of almost four-fifths of patients who were taking beta-blocker therapy at time of onset had the expected two peaks of symptoms onset, but higher evening peaks, with statistically different distribution in the group of patients undergoing beta-blocker therapy. These subgroups of patients, together with subgroups of patients with dominant evening peaks (patients with CHF and patients with non-STEMI) and patients with no peak (patients with previous angina and previous AMI), probably appear to modify characteristic circadian variation of infarction onset, expressing a higher evening peak, respectively to the previous CABG, with adverse consequences for central nervous system functioning.

## ACKNOWLEDGMENTS

This paper is not under consideration elsewhere. None of the paper's contents have been previously published. All authors have read and approved the manuscript. There is no potential conflict of interest.
